# Enhanced Supercapattery Performance Enabled by Nitrogen-Doped Nb_2_O_5_ Nanostructures

**DOI:** 10.1021/acsomega.5c07882

**Published:** 2025-11-26

**Authors:** Fernando José Soares Barros, Samuel da Silva Eduardo, Klebson Lucas Pereira Cardozo, Hector A. Vitorino, Carlos Martins Aiube, Mariana Lumi Ichihara Sado, Camila de Lima Ribeiro, Alysson Martins Almeida Silva, Francisco Murilo Tavares de Luna, Auro Atsushi Tanaka

**Affiliations:** a Department of Chemistry, 37892 Federal University of Maranhão, Av. dos Portugueses, 1966, São Luís, MA 65080-805, Brazil; b Department of Chemical Engineering, 28121 Federal University of Ceará, Campus do Pici, 709, Fortaleza, CE 60455-760, Brazil; c Department of Chemistry, Universidade Federal do Rio de Janeiro, Avenida Athos da Silveira Ramos, no. 149, Rio de Janeiro, RJ 21941-909, Brazil; d Department of Fundamental Chemistry, Institute of Chemistry, 28133 University of São Paulo, Av. Prof. Lineu Prestes, 748, São Paulo, SP 05508-000, Brazil; e Institute of Chemistry, 28127 University of Brasília, Campus Universitário Darcy Ribeiro, Asa Norte, Brasília, DF 70910-900, Brazil; f Department of Mechanical Engineering, 28127 University of Brasília, Campus Universitário Darcy Ribeiro, Asa Norte, Brasília, DF 70910-900, Brazil

## Abstract

Nitrogen doping has emerged as a strategy to enhance the electrochemical performance of transition metal oxides. In this work, nitrogen-doped niobium oxide (Nb_N) was synthesized via a thermal treatment using urea as the nitrogen precursor. Structural and compositional analyses of Nb_N confirmed successful nitrogen incorporation without phase changes, leading to improved crystallinity and larger crystallites, with XPS revealing increased oxygen vacancies and Raman analysis indicating local lattice distortion. Electrochemical performance was evaluated in a 2 M KOH electrolyte. Cyclic voltammetry and galvanostatic charge–discharge tests revealed a battery-type electrode behavior with higher specific capacity values at 1 A g^–1^ for Nb_N (1297.37 C g^–1^) compared to the undoped Nb_U (1108.75 C g^–1^). Moreover, Nb_N demonstrated rate capability and superior cycling stability. A supercapattery assembled with Nb_N as the positive electrode and activated carbon as the negative electrode delivered an energy density of 496.65 Wh kg^–1^ and a power density of 2771.99 W kg^–1^, with remarkable stability over 5000 cycles. Nitrogen doping enhanced the structural and electronic properties of Nb_2_O_5_, improving its pseudocapacitive behavior and making it suitable for high-performance supercapatteries.

## Graphical abstract

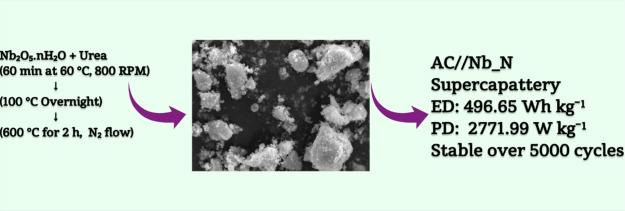

## 1 Introduction

The growing need for cleaner power has accelerated the adoption of technologies such as solar and wind. Their generation, however, is variable because it depends on weather conditions. Storage systems mitigate this by retaining surplus electricity and supplying it during shortfalls, ensuring more stable output. In view of climate concerns and the impact of fossil fuels, combining renewables with effective storage is essential for a sustainable energy future.^1,2^

Energy storage devices include batteries, capacitors, and supercapacitors, each with distinct characteristics.^3,4^ Among these, the concept of a “supercapattery” has emerged to unify hybrid systems that combine the attributes of supercapacitors and batteries.^6^ Supercapatteries leverage both capacitive and noncapacitive Faradaic charge storage mechanisms, making them distinct from traditional devices. This unified term encompasses a range of hybrid devices previously referred to as redox capacitors, Li-ion capacitors, Na-ion capacitors, hybrid electrochemical capacitors, battery–supercapacitor hybrids, and pseudocapacitors.^5,6^

Recent efforts have focused on transition metal oxides as electrode materials due to their multifunctional properties.^7,8^ Amonng them, nanostructured ruthenium oxide,^9^ manganese oxide,^10^ spinel ferrites,^11^ and cobalt oxide^12^ have been extensively investigated for supercapatteries.^12^ Niobium and its oxides have attracted attention in fields ranging from catalysis to energy storage. Nb-based materials exhibit excellent performance in photocatalysis and as solid acid catalysts, with Nb_2_O_5_ demonstrating considerable catalytic activity for water decomposition and organic transformations.^13^ In electrochemical energy storage, Nb_2_O_5_ has been applied in both supercapacitors and lithium/sodium-ion batteries due to its intercalation capacity and pseudocapacitive behavior.^14,15^ However, its low electrical conductivity and sluggish ion diffusion in the bulk phase limit practical applications.^16^ Researchers have investigated several approaches, such as incorporating Nb_2_O_5_ into carbon-based materials like graphene and carbon nanotubes, to enhance its properties.^15,16^

Building on these efforts, nitrogen doping has emerged as another effective route to tailor material properties, enhancing their functionality in catalysis,^17^ photocatalysis,^18^ and supercapacitors.^19^ Nitrogen-doped carbon, for instance, has been widely explored for oxygen reduction reactions (ORR), often outperforming traditional metal-based catalysts.^20^ Extending this concept to niobium oxides, nitrogen-doped Nb_2_O_5_ has demonstrated improved visible-light absorption, leading to higher efficiency in photocatalytic hydrogen production.^18^ For energy storage materials, nitrogen-doped porous carbon offered high specific surface areas and pseudocapacitive behavior.^21^ Different nitrogen precursors, including urea,^18,21^ phenanthroline,^20^ and dicyandiamide,^19^ have been employed to fine-tune these properties for specific applications.

Niobium oxides have been studied for energy storage, but their capacitance and cycling stability remain limited. Nitrogen doping has the potential to improve these properties, yet its effects in supercapattery devices are not fully understood. In this work, we present a simple and scalable method to synthesize nitrogen-doped Nb_2_O_5_ using urea under a nitrogen atmosphere. We aim to investigate how nitrogen incorporation influences the structure, defects, and electrochemical performance in a supercapattery device.

## 2 Experimental Section

### 2.1 Preparation of the Electrode Material

The Nb-based nanomaterial was prepared by a simple thermal method. Briefly, niobium oxide hydrate (Nb_2_O_5_·*n*H_2_O) (HY-340, CBMM) was calcined at 600 °C for 3 h without N_2_ flow at a rate of 5 °C/min. This material was labeled as Nb_U.

The doping of the Nb_U electrode material with nitrogen was based on the method proposed elsewhere.^17^ First, 0.5 mmol of Nb_2_O_5_·*n*H_2_O and 10 mmol of urea (Dinâmica, Brazil) were stirred in 100 mL of ethanol (Dinâmica, Brazil) for 60 min at 60 °C, 800 rpm. The solid was separated and dried overnight at 100 °C. Next, the material was calcined at 600 °C for 2 h under N_2_ flow with a heating rate of 25 °C/min. Such an atmosphere promotes the incorporation of nitrogen species released during urea decomposition into Nb_2_O_5_.^22^ The material was labeled as Nb_N.

### 2.2 Measurements and Characterization

The materials were assessed using X-ray diffraction (XRD) with a Rigaku MiniFlex diffractometer from Japan, utilizing Cu Kα radiation (λ = 1.5406 Å) across a 2θ range of 10–80° at a scanning speed of 2°/min. The crystallite size was derived from the Debye–Scherrer formula, based on the (110) diffraction plane, as follows:^6^

$$d=\frac{K\times \lambda }{\beta \times \cos\;\theta }$$ 1

Here, *d* denotes the crystallite size, *K* is the shape factor (0.9), λ represents the X-ray wavelength, β is the peak full width at half-maximum (fwhm), and θ is the Bragg angle.

The dislocation density (ρ_d_) was calculated from the crystallite size (*d*), using the following relation:^23^

$$\rho_{d}=\frac{1}{d^{2}}$$ 2

The mean lattice strain (ε) was estimated using the relation:

$$\varepsilon =\frac{\beta \times \cos\;\theta }{4}$$ 3

Inductively coupled plasma–optical emission spectrometry (ICP-OES) was conducted using a Spectro Arcos device (SPECTRO Analytical Instruments GmbH, Kleve, Germany) with a detection limit of 0.01 ppm. The sample digestion method consisted of alkaline fusion with lithium tetraborate followed by dissolution with a 1:1 mixture of nitric acid and hydrofluoric acid.

The elemental composition was determined using a carbon–hydrogen–nitrogen (CHN) analyzer (PerkinElmer 2400, USA) equipped with a thermal conductivity detector (TCD). The morphology of the samples was examined using a field emission scanning electron microscope (JEOL JSM 7100F, Japan), which includes an electron dispersive X-ray spectroscopy system (EX-37270VUP).

For transmission electron microscopy (TEM) studies, a JEOL JEM-2100 was employed. Sample preparation for TEM involved suspending the nanostructures in isopropyl alcohol, followed by ultrasonic agitation and deposition of a drop onto a TEM Cu grid with an amorphous holey lacey film.

The thermal stability of the samples was assessed by thermogravimetric analysis in SKIMMER (Netzsch, Germany) equipment, under synthetic air with a heating ramp of 10 °C/min in a range from 30 to 1000 °C.

Confocal Raman spectra for Nb_U and Nb_N were acquired using an Alpha 300 system from Witec (Ulm, Germany) equipped with a highly linear (0.02%) piezo-driven stage, a 100×/NA = 0.90 Nikon objective, and with laser excitation of HeNe (632.8 nm), argon (488 nm), and Nd:YAG (532 nm).

X-ray photoelectron spectroscopy (XPS) was performed using a K-Alpha spectrometer from Thermo Fisher (East Grinstead, United Kingdom) with monochromatic Al Kα (*h*ν = 1486.6 eV) radiation as the excitation source. High-resolution spectra were recorded with a pass energy of 0.05 eV. For each element, 20–30 scans were accumulated to enhance the signal-to-noise ratio.

### 2.3 Electrochemical Measurements

Electrochemical measurements were conducted using a potentiostat model Autolab PGSTAT302N (Metrohm, The Netherlands), with data processed via NOVA 2.1.4 software. Experiments utilized a three-electrode configuration, comprising a Ag/AgCl reference electrode, a platinum wire counter electrode, and a working electrode made of nickel foam coated with the active material (area = 1.5 cm^2^). For the working electrode, nickel foams were cut into squares of 1.5 cm^2^ and pretreated in 1 M HCl solution for 1 h. They were then rinsed with deionized water using an ultrasonic bath for 15 min (repeated three times) and dried in an oven at 60 °C. The electroactive slurry was prepared by mixing the active material, Super P, and a PVDF/NMP solution (1.5% w/v) in an 8:1:1 ratio, with the PVDF solution added dropwise during mixing in an agate mortar and pestle for 10 min. Subsequently, 20 μL of the suspension was drop-cast onto the cleaned Ni foam using a micropipette. A nickel rod was then attached to the foam to serve as the current collector. The Ni foam substrate was mechanically pressed after deposition to promote intimate contact between the active layer and the current collector. This procedure is widely used in supercapacitor electrode preparation, as it enhances electrical conductivity and adhesion while preserving the mechanical integrity of the foam.^24,25^ Finally, the electrode was dried overnight at 60 °C. The mass of the active material effectively deposited was determined by weighing the foams before and after impregnation. A 2 M KOH solution was used as the electrolyte.

The electrochemical behavior was evaluated using cyclic voltammetry (CV) at scan rates of 5, 10, 20, 40, and 80 mV s^–1^, within a potential range of −0.2 to 0.5 V, thus covering the entire electrochemical stability region of the electrolyte. Galvanostatic charge–discharge experiments were performed at current densities ranging from 1 to 20 A g^–1^. The supercapacitor cycling stability was assessed over 1000 charge–discharge cycles, and electrochemical impedance spectroscopy (EIS) measurements were conducted before and after cycling. EIS data were collected across a frequency range of 0.1–1000 Hz.

The specific capacity of the electrode material in C g^–1^ was calculated with the data from the GCD discharge curves and eq 4:

$$Q=\frac{I\times \Delta t}{m}$$ 4

Here, *Q* is specific capacitance, *I* is the current, Δ*t* in s is the discharge time, and *m* is the mass of the electrode material in mg.

To allow comparison with recent Nb_2_O_5_-based supercapacitor studies, the same data were also used to express the electrochemical performance as specific capacitance in F g^–1^, according to eq 5:^26^

$$C_{s}=\frac{I\times \Delta t}{\Delta V\times m}$$ 5

where *C*_s_ is specific capacitance and Δ*V* in V represents the potential drop.

The Coulombic efficiency (η) was calculated with eq 6:^27^

$$\eta =\frac{t_{d}}{t_{c}}\times 100\%$$ 6

where *t*_d_ is the discharging time and *t*_c_ is the charging time.

The supercapaterry test was performed in a configuration of two electrodes, one working electrode containing the active material and the other with activated carbon. The preparation of both electrodes followed the same procedure mentioned above. As an initial step, to calculate the required amount of activated carbon in the electrode for the supercapattery test, the electrochemical tests were performed with a carbon electrode in the three-electrode configuration, having a similar value of mass to the electrode with the active material. From this test, obtained values of current and specific capacitance were used alongside those of the active material and the following mass/charge (eq 7):^27^

$$\frac{m+}{m-}=\frac{Q+}{Q-}=\frac{m\times C_{s}\times \Delta V}{m\times C_{s}\times \Delta V}$$ 7

where *m*+/– and *Q*+/– mean the mass and charge balance, respectively, *m* is the mass (in mg), *C*_s_ is specific capacitance, and Δ*V* in V represents the potential drop of both the activated carbon and the active material.

For energy storage devices, energy density (ED) and power density (PD) are very relevant parameters; they were calculated with eqs 8 and 9, respectively:^26^

$$\mathrm{ED}=\frac{1}{2}\times C_{s}(\Delta V{)}^{2}$$ 8

$$\mathrm{PD}=3600\times \frac{\mathrm{ED}}{\Delta t}$$ 9

where *C*_s_ is the specific capacitance (F g^–1^) obtained in the GCD tests for the hybrid supercapacitor, Δ*V* (V) is the potential range between the cathode and anode, and Δ*t* (s) is the time of discharge of the device. All the tests were carried out at room temperature.

The GCD experiments were performed at current densities ranging from 1 to 50 A g^–1^. The supercapattery cycling stability was assessed over 10,000 charge–discharge cycles.

## 3 Results and Discussion

### 3.1 Characterizations of Electrode Materials

The composition of the solids was analyzed using ICP-OES and CHN analysis. The Nb content was measured by ICP-OES, yielding 67.63% for the calcined Nb_2_O_5_·*n*H_2_O (Nb_U) and 69.40% for the nitrogen-doped material (Nb_N). In the CNH elemental analysis, the precursor HY-340 contained 0.17% C, 1.15% H, and 0.08% N, whereas its calcined form (Nb_U) showed reduced levels of 0.13% C, 0.48% H, and 0.03% N. This decrease, particularly in hydrogen and nitrogen, reflects the removal of water and volatile residues during calcination. Following nitrogen doping with urea, Nb_N exhibited 1.35% C, 0.28% H, and an increase to 1.96% N, indicating nitrogen incorporation and residual carbon from urea decomposition.

To evaluate the structural impact of nitrogen doping, XRD analyses were performed on the precursor Nb_2_O_5_·*n*H_2_O (HY-340), its calcined form (600 °C for 3 h), the undoped sample (Nb_U), and the nitrogen-doped sample (Nb_N) (Figure 1 and Table 1). The pristine HY-340 precursor exhibits a broad diffuse signal, confirming its amorphous nature.^28,29^ After calcination at 600 °C without N_2_ flow, the Nb_U sample shows sharp diffraction peaks indexed to the orthorhombic Nb_2_O_5_ phase (JCPDS 27-1003), indicating the successful crystallization of the oxide.^30^ The presence of dopant nitrogen in Nb_N leads to a similar diffraction pattern, but with slightly higher peak intensity and reduced peak broadening, suggesting improved crystallinity and possible modification of the lattice structure. The main reflections observed at 26.4, 33.3, 43.0, 54.3, 59.7, and 65.2 correspond to the (001), (180), (181), (002), (380), and (212) crystal planes in agreement with the reference pattern.^30^

**Figure 1:**
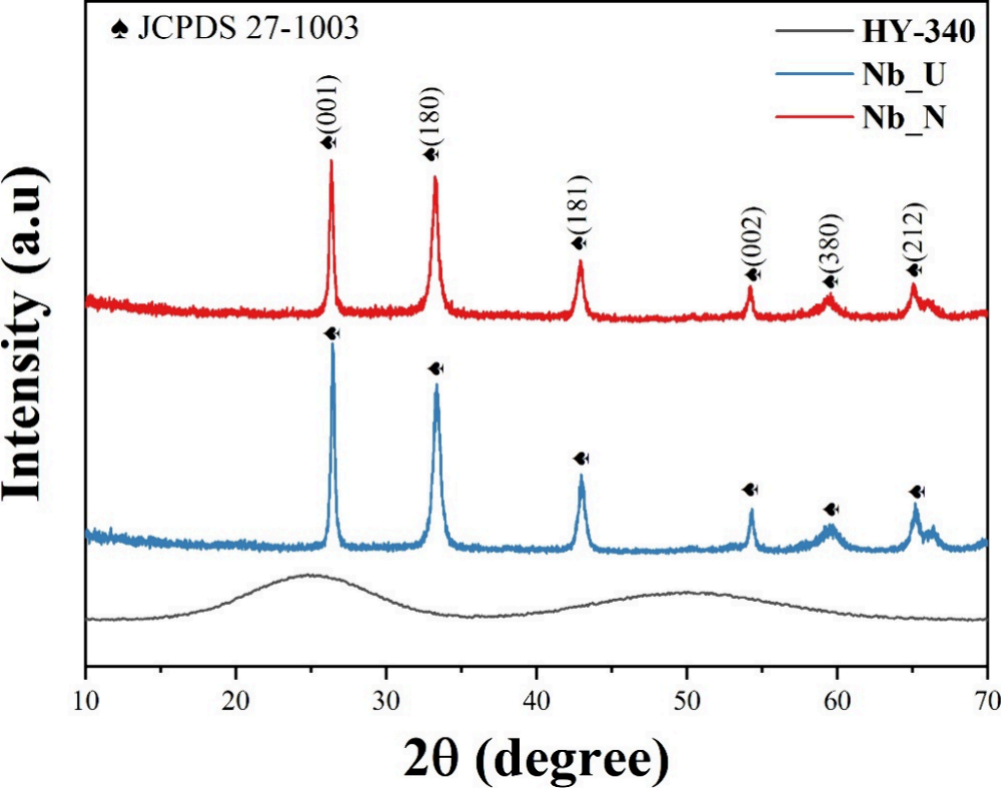
XRD results for HY-340, Nb_U, and Nb_N.

**Table 1:** XRD data for Nb_U and Nb_N

		lattice parameters (Å)					
sample	*d* (nm)	*a*	*b*	*c*	*V* (Å^3^)	ε	ρ_d_ (m^–2^)
Nb_U	33.09	6.1977	26.3702	3.9362	643.31	0.00463	9.35 × 10^14^
Nb_N	66.16	6.2046	26.6898	3.9457	653.41	0.00233	2.34 × 10^14^

The diffractograms show that both materials consist mainly of crystalline phases with high purity. Nitrogen incorporation does not generate new phases but slightly modifies the orthorhombic structure, as reflected in the structural parameters in Table 1.The crystallite size increases from 33.09 nm for Nb_U to 66.16 nm for Nb_N, indicating significant grain growth during the nitrogen doping. In addition, the unit cell volume expands slightly from 643.31 to 653.41 Å^3^, suggesting minor lattice distortions associated with the presence of the dopant. The microstrain decreases (ε) from 0.00463 to 0.00233, while the dislocation density drops from 9.35 × 10^14^ to 2.34 × 10^14^ m^–2^, evidencing improved crystallinity and reduced defect concentration.^31^

The TEM images in Figure 2 provide detailed insights into the morphology and microstructure of Nb_U (A, B) and Nb_N (C, D). For calcined Nb_2_O_5_·*n*H_2_O (Nb_U), Figure 2A shows agglomerates of Nb_2_O_5_ particles with an average size below 200 nm at low magnification, while Figure 2B reveals lattice fringes indicative of a well-crystallized structure, with an interplanar spacing of approximately 0.39 nm, consistent with the (001) plane of orthorhombic Nb_2_O_5_ (JCPDS PDF no. 27-1003). In the case of nitrogen-doped Nb_2_O_5_ (Nb_N), Figure 2C displays similar agglomerates to the calcined material, and Figure 2D shows lattice fringes with slight distortions compared to the undoped Nb_2_O_5_, likely due to nitrogen incorporation into the Nb_2_O_5_ lattice, introducing local structural disorder. This is consistent with the XRD results, which confirm the preservation of the orthorhombic Nb_2_O_5_ phase but with sharper and more intense reflections, indicating enhanced crystallinity upon doping.

**Figure 2:**
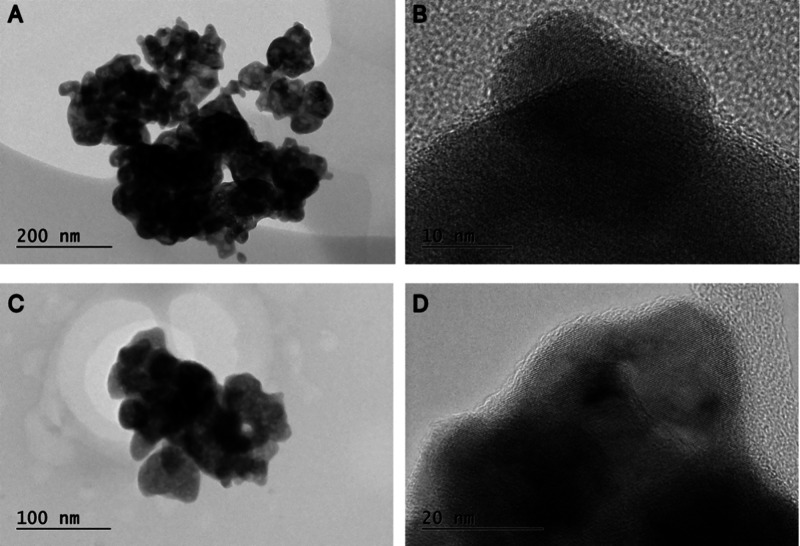
TEM images for Nb_U (A, B) and Nb_N (C,D).

While TEM images provided detailed information on crystallinity and interlayer spacing at the nanoscale, SEM (Figure 3) offers complementary insights into the particle morphology and size distribution. The SEM image of Nb_U (Figure 3A) reveals particles with a predominantly irregular and agglomerated morphology. A heterogeneous size distribution is evident, with larger clusters surrounded by smaller particles. The larger aggregates tend to exhibit rounded but still irregular contours, whereas some of the smaller particles display more angular edges. Energy-dispersive X-ray (EDS) analysis was conducted to further examine the compositional differences between the angular and round particles, as shown in Figure 3B,C. The corresponding color maps illustrate the uniform distribution of Nb, O, and N across the particles, serving as qualitative confirmation rather than quantitative ratios. According to the ED spectra, the angular particles exhibited Nb and O mass percentages of 67.88 and 32.12%, respectively, while the round particles showed 55.34% Nb and 44.66% O (Figure S1).

**Figure 3:**
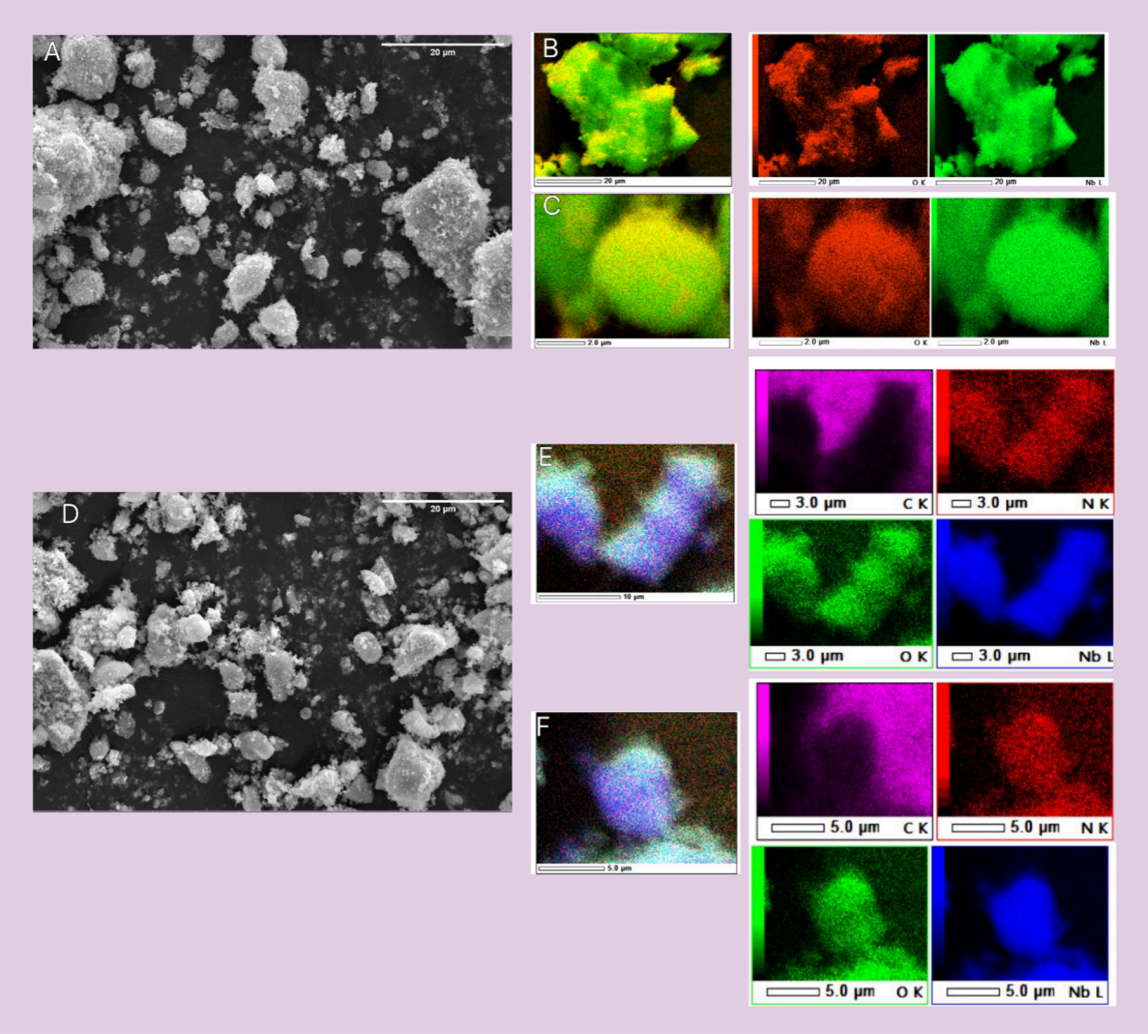
SEM images and EDS elemental mapping of undoped Nb_2_O_5_ (Nb_U) and nitrogen-doped Nb_2_O_5_ (Nb_N). (A–C) Nb_U: overview SEM micrograph (A) with corresponding EDS mappings of angular (B) and round (C) particles, showing Nb and O. (D–F) Nb_N: overview SEM micrograph (D) with EDS mappings of angular (E) and round (F) particles, showing Nb, O, C, and N.

For nitrogen-doped Nb_2_O_5_ (Nb_N), Figure 3D also shows particles with a variety of shapes and sizes. Among these, the round particles appear more spherical, indicating that their edges may have been smoothed during the synthesis process. This morphology is likely the result of surface diffusion during calcination, possibly assisted by gas evolution from urea decomposition, which promotes isotropic growth and surface energy minimization.^32^ Thus, the particles exhibit smoother and more rounded surfaces. In contrast with Nb_U, after nitrogen doping, the angular particles have sharper edges and well-defined corners. These shapes suggest that the particles might have been formed through a brittle fracture process or through crystallization in a manner that preserves the original angularity of the crystal faces. EDS analysis, as illustrated in Figure 3E,F, reveals a uniform distribution of nitrogen across both angular and round particles. Since urea was used as the dopant precursor, the sample was analyzed for C and N. The elemental maps revealed that C was not associated with the particles but was detected in the background, likely from the carbon tape used during sample preparation. Therefore, C was excluded from the elemental quantification, and only Nb, O, and N were considered. EDS spectra (Figure S1) show that the angular particles contain 46.02% Nb, 48.59% O, and 5.39% N, whereas the round particles comprise 43.63% Nb, 55.12% O, and 3.88% C. In agreement with a previous report,^33^ EDS mapping confirmed incorporation of nitrogen within Nb_2_O_5_ particles, supporting the qualitative uniform distribution observed in this work. Guo et al. used urea as the nitrogen precursor for doping, demonstrating that this strategy can enhance the photocatalytic activity of Nb_2_O_5_ without altering its overall morphology^33^

The Raman spectra of Nb_U and Nb_N (Figure 4) reveal not only the vibrational modes associated with the Nb_2_O_5_ lattice^34^ but also the influence of particle morphology on the structural features. For Nb_U (Figure A), both round and angular particles display the characteristic modes of orthorhombic Nb_2_O_5_ at ∼95, 245, and 693 cm^–1^, in agreement with the XRD confirmation of phase formation after calcination. However, the angular particles show a relatively higher intensity and sharper peaks, suggesting improved structural ordering compared to the round ones, which display broader features indicative of more disordered domains.

**Figure 4:**
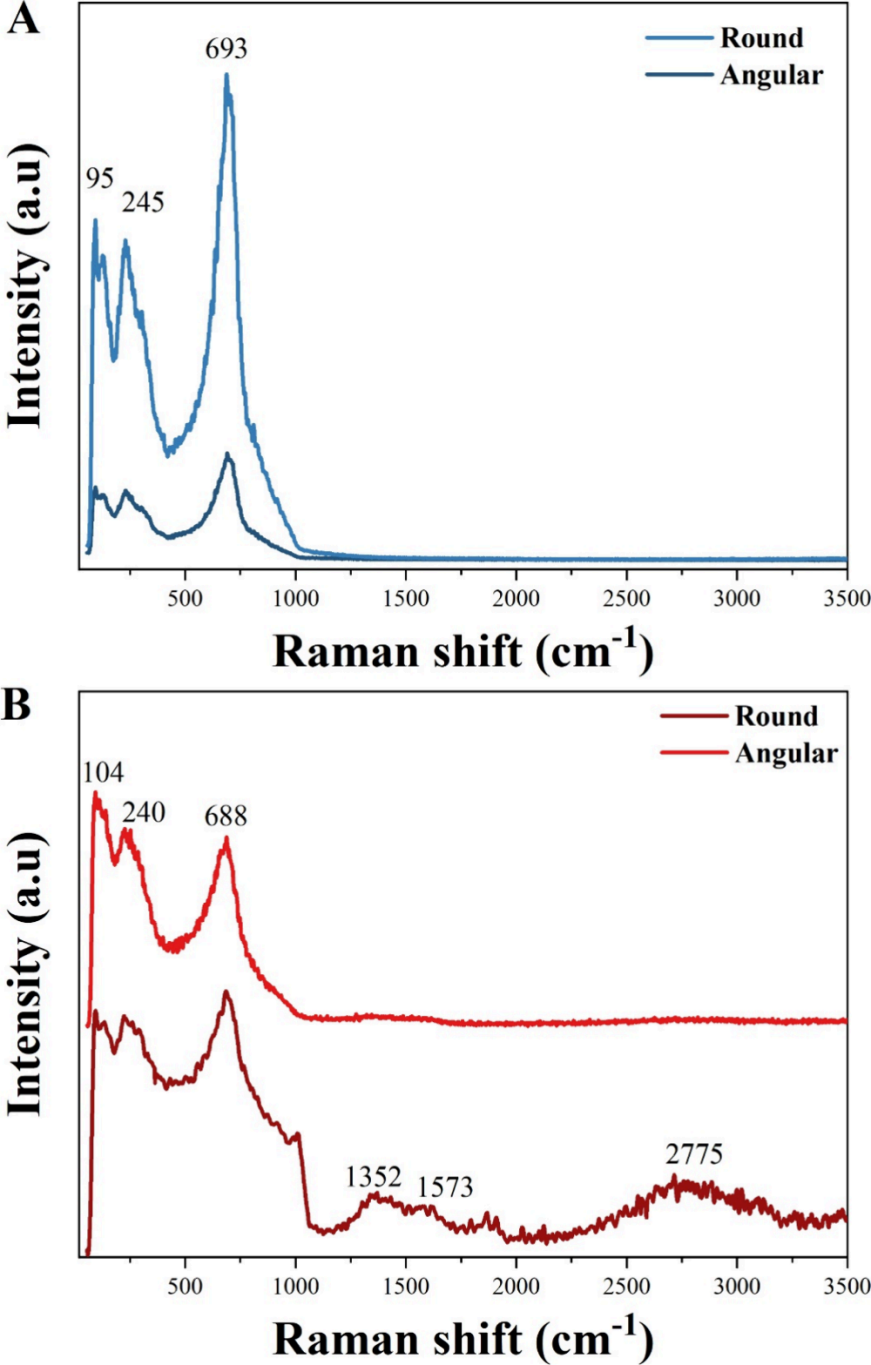
Raman spectra of round and angular partices for Nb_U (A) and Nb_N (B).

For Nb_N (Figure 4B), the Raman spectra display similar vibrational modes to Nb_U, but with broader features, particularly in the angular particles. This broadening contrasts with the XRD results, where nitrogen-doped Nb_2_O_5_ retained the orthorhombic phase yet showed sharper reflections, suggesting that while long-range crystallinity was improved, local distortions arose from nitrogen incorporation and were more evident in Raman spectra. The main Nb–O modes at 104, 240, and 688 cm^–1^ are preserved in both morphologies, but the angular particles exhibit additional bands at higher wavenumbers, indicating a stronger contribution from nitrogen species. These spectral differences align with SEM observations (Figure 3), which revealed distinct particle morphologies.

The Raman spectra for round Nb_N particles also showed peaks at ∼1350 cm^–1^ (D band) and ∼1580 cm^–1^ (G band), typical of disordered and graphitic carbon. A peak at ∼2700 cm^–1^, corresponding to the carbon 2D band, further supports this.^35,36^ Although EDS analysis (Figure 3E,F) did not detect carbon in the particle itself, the Raman sensitivity to trace amounts confirmed carbon in graphitic forms. A likely origin for these features is the partial carbonization or formation of N-containing carbonaceous products from the thermal decomposition of urea during the N_2_ calcination step. The presence of carbon in some particles of Nb_N is not detrimental, as carbon species are often reported to improve conductivity and electrochemical performance in niobium-based materials. For example, ultrasmall Nb_2_O_5_ nanoparticles with N-doped carbon exhibited improved pseudocapacitive behavior and faster reaction kinetics due to enhanced electron transport and reduced ion–electrode diffusion paths.^37^ Also, carbon-coated ultrathin T-Nb_2_O_5_ nanosheets maintain high capacity retention at elevated current densities, attributed to the carbon layer enabling better conductivity and structural integrity.^38^

Thermogravimetric analysis results are presented in Figure 5. In the precursor, Nb_2_O_5_·*n*H_2_O (HY-340), a marked weight loss related to the loss of adsorbed water, occurs around 100 °C. The weight stabilizes at 83% by 1000 °C, in accordance with the moisture information (∼20% w/w) supplied for HY-340.^28^ For the calcined solid, Nb_U, a minimal weight loss is observed throughout the heating range, typical of orthorhombic Nb_2_O_5_.^39^ The weight remains 99.8% at 1000 °C, consistent with the high thermal stability of metal oxides. In the nitrogen-doped solid Nb_N, prepared using urea as the nitrogen precursor, the presence of amine groups (−NH_2_) leads to a slight weight loss, with a more pronounced decline beginning around 600 °C. The weight then stabilizes at 96.6% by 1000 °C. Accordingly, the TGA results imply that the Nb_N structures consist of 3.21 wt % of C and N, in agreement with the elemental analysis.

**Figure 5:**
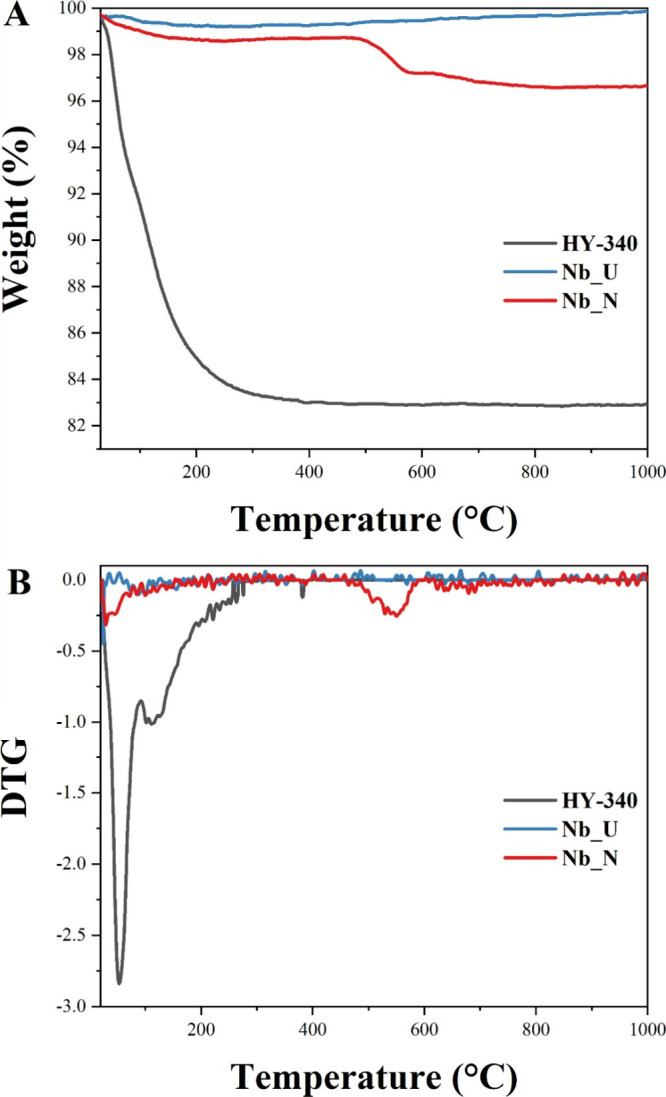
Thermogravimetric and derivative analyses for HY-340, Nb_U, and Nb_N: TGA (A) and DTG (B).

For HY-340, the DTG curve (Figure 5B) shows the major peak around 100–200 °C, indicating a rapid weight loss, likely due to the release of adsorbed water and volatile species.^28^ Smaller peaks are observed up to 800 °C. The DTG curve of Nb_U solid is relatively flat with minimal fluctuations, suggesting negligible decomposition and high thermal stability. The DTG curve for Nb_N shows minor fluctuations, particularly around 600–700 °C, ascribed to nitrogen and carbon content decomposition.^40^ The minimal weight loss and low decomposition rates of Nb_U and Nb_N indicate that these materials can operate efficiently at elevated temperatures without considerable degradation.

The elemental surface compositions of undoped and N-doped electrode materials were assessed using X-ray photoelectron spectroscopy (XPS). For Nb_U (Figure 6A), high-resolution spectra exhibit two distinct peaks in the Nb 3d region, characteristic of the spin–orbit doublet of niobium: Nb 3d_5/2_ at approximately 207.9 eV and Nb 3d_3/2_ at around 210.6 eV. These binding energies are consistent with Nb^5+^ in Nb_2_O_5_, indicating that Nb_U has niobium in its fully oxidized state.^19,41^ In the O 1s spectrum, the main peak is observed at ∼530.9 eV. While slightly higher than the typical B.E. for pure lattice oxygen (commonly ∼530.0–530.3 eV),^16,40,41^ this value is attributed to Nb–O bonds within the Nb_2_O_5_ lattice.^42^ The peak, at ∼532.4 eV, represents surface-adsorbed oxygen (O_ads_), from oxygen vacancies.^43–45^

**Figure 6:**
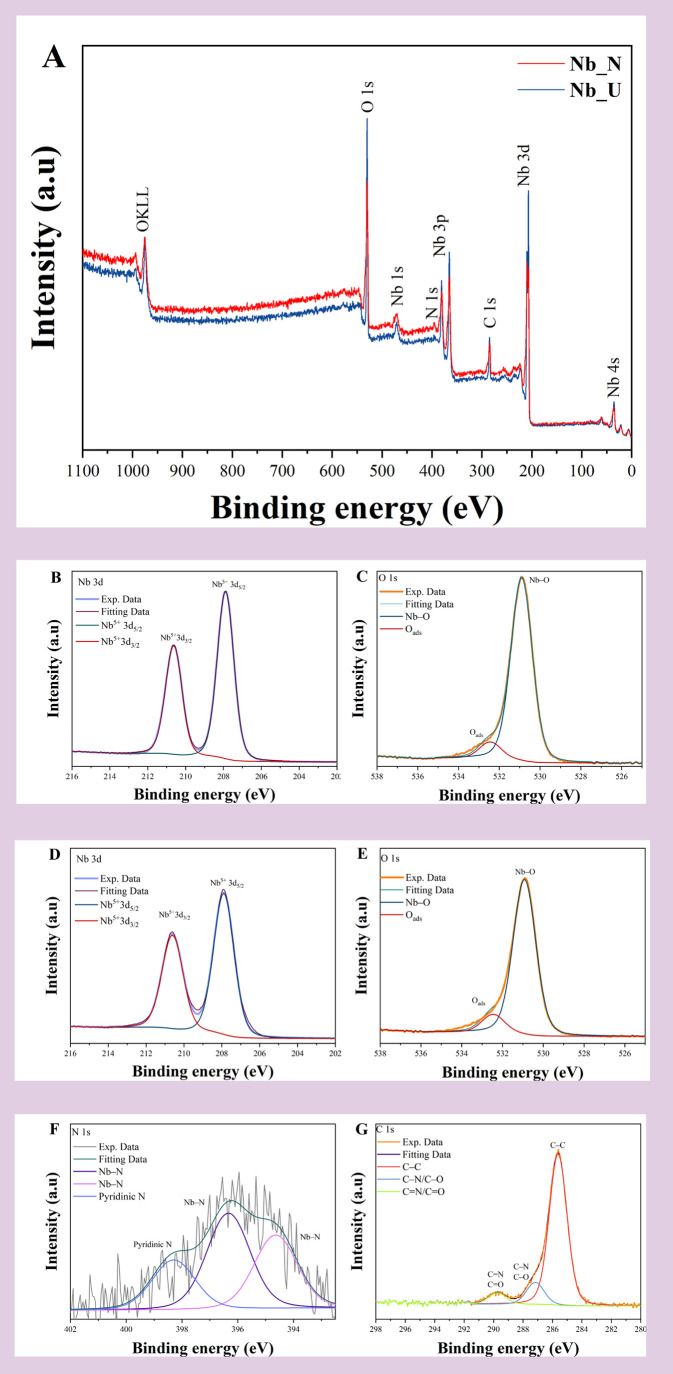
XPS survey for Nb_U and Nb_N. (A) High-resolution XPS spectra for Nb_U Nb 3d (B) and O 1s (C); for Nb_N Nb 3d (D), O 1s (E), N 1s (F), and C 1s (G).

The XPS analysis reveals distinct chemical and electronic modifications in the nitrogen-doped Nb_2_O_5_ (Nb_N) sample compared to the undoped material. Both Nb_U and Nb_N display well-defined Nb^5+^ peaks at ∼207.9 eV (Nb 3d_5/2_) and ∼210.6 eV (Nb 3d_3/2_) (Figure 6C), indicating the presence of Nb_2_O_5_. However, a slight increase in the relative intensity of the Nb 3d_3/2_ peak in the Nb_N sample suggests a modification in the electronic environment of niobium. The substitution of oxygen by nitrogen in Nb_2_O_5_ leads to increased electron density around Nb atoms, potentially altering the 3d binding energy and the relative abundance of spin–orbit components.^18^ In the O 1s spectrum (Figure 6D), both samples exhibited a peak at ∼530.9 eV, typical of lattice O^2–^ in Nb_2_O_5_, but the Nb_N sample also presents a more intense shoulder at ∼532.5 eV (O_ads_), suggesting that nitrogen incorporation promotes the formation of lattice oxygen vacancies.^6,46^

The N 1s spectrum (Figure 6E) confirms the incorporation of nitrogen into the Nb_2_O_5_ lattice. The main peak at ∼396.3 eV is assigned to substitutional nitrogen forming Nb–N bonds,^19,41^ while the component at ∼398.3 corresponds to pyridinic-like nitrogen species.^17^ The third peak at ∼394.6 is also likely associated with substitutional nitrogen atoms, pointing to the formation of Nb–N bonds within the oxide framework.^33,47^ For the C 1s region (Figure 6F), the peak observed at ∼284.8 eV corresponds to C–C bonds, attributed to adventitious carbon contamination from the environment.^48^ The peak located at ∼287.2 eV is related to C–N and/or C–O bonds, indicating the incorporation of nitrogen species into the material, likely through the formation of C–N linkages as a result of urea decomposition.^19,49^ The third peak at ∼289.8 eV is associated with C=N and C=O bonds, related to the presence of nitrogen-containing functionalities and residual oxygenated species.^49^ These features reflect the complex surface chemistry introduced by nitrogen doping and suggest potential modifications to the electronic environment of Nb_2_O_5_, which may enhance its electrochemical performance.

### 3.2 Electrochemical Performance

The electrochemical performance of calcined Nb_2_O_5_·*n*H_2_O (Nb_U) and nitrogen-doped (Nb_N) electrode materials, deposited on nickel foam, was investigated using cyclic voltammetry (CV) analysis in an alkaline solution (2 M KOH) (Figure 7). The active mass deposited on the electrodes was 0.0011 g for Nb_U and 0.0021 g for Nb_N. Measurements were performed over a wide range of potentials, ranging from −0.2 to 0.5 V versus Ag/AgCl.

**Figure 7:**
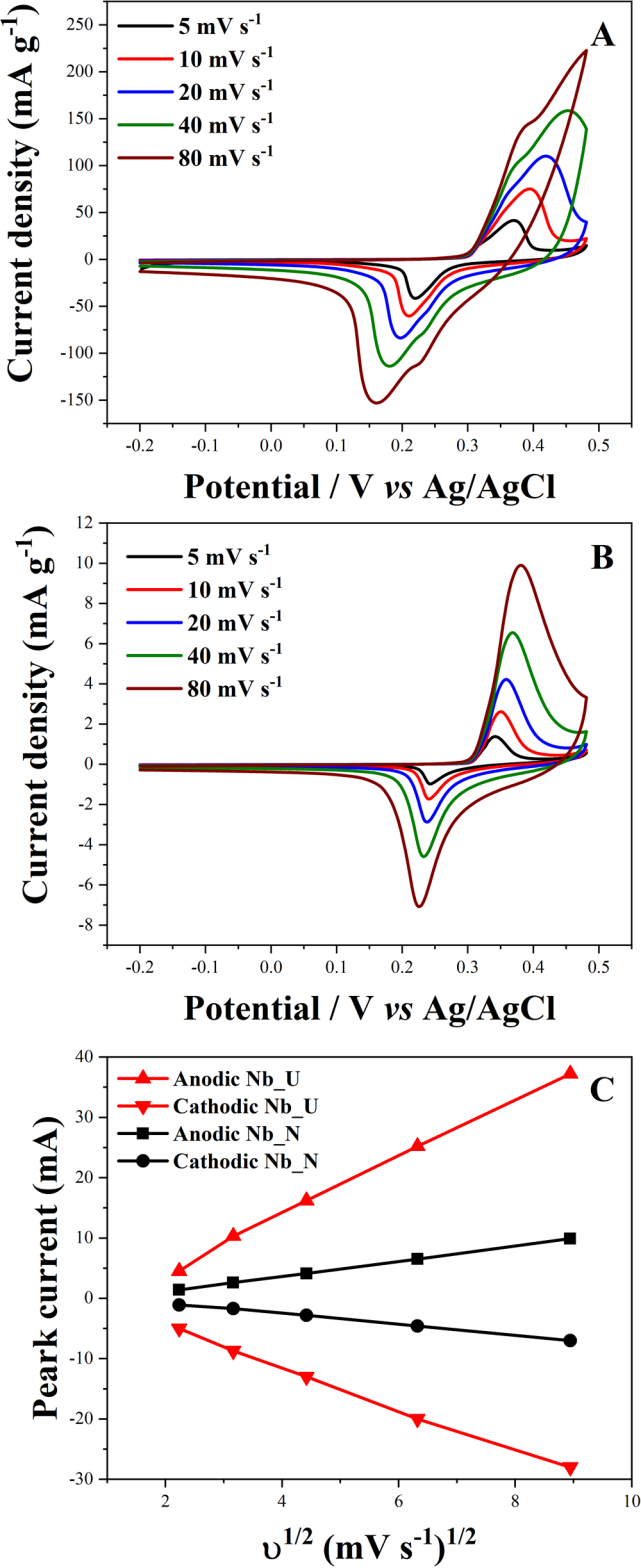
CV for a three-electrode configuration for Nb_U (A) and Nb_N (B). Peak current versus the square root of the sweep speed υ^1/2^ (C).

The cyclic voltammetry technique allowed the systematic evaluation of electrochemical responses, revealing the performance of both materials at different scan rates (5, 10, 20, 40, and 80 mV s^–1^). The CV results, shown in Figure 7A,B, show a pseudocapacitance behavior in the Nb_U and Nb_N electrodes. This characteristic is attributed to the redox reactions that occur between the material and the electrolyte during the electrochemical process.^20,21,50,51^ Nb_N shows stable CV profiles across scan rates, confirming good rate capability, while Nb_U displays distortions at higher currents, indicating lower stability under fast charge–discharge conditions.

The observed pseudocapacitance suggests high charge storage capacity in these electrodes, going beyond the mere formation of an electrical layer at the solid–liquid interface. The dynamic interaction between Nb_U and Nb_N with the electrolyte in the KOH solution leads to an electrochemical response as pseudocapacitance, indicating the formation and reversibility of redox reactions during sweep cycles.^52^

Considering XPS results for Nb_U, the redox behavior in KOH is likely associated with a surface Nb^5+^/Nb^4+^ transition facilitated by OH^–^ adsorption:^53^

$${\mathrm{Nb}}_{2}O_{5}+x{\mathrm{OH}}^{-}+xe^{-}↔{\mathrm{Nb}}_{2}O_{5}(\mathrm{OH}{)}_{x}$$ 10

The OH^–^ ions from the 2 M KOH electrolyte adsorb onto the surface and participate in the pseudocapacitive reaction.

With nitrogen doping, the mechanism for Nb_N is similar, but oxygen vacancies and nitrogen sites modify the surface reactivity. The oxygen vacancies and N sites can serve as additional active centers facilitating OH^–^ adsorption/desorption. The redox reactions are more reversible due to these defects, improving the ionic/electronic transport. Thus, for Nb_N,

$${\mathrm{Nb}}_{2}O_{5}:N+x{\mathrm{OH}}^{-}+xe^{-}↔{\mathrm{Nb}}_{2}O_{5}:N(\mathrm{OH}{)}_{x}$$ 11

Simultaneously, nitrogen doping can improve the electronic conductivity of the material, promoting faster charge transfer during cycling. Such synergistic effects between oxygen vacancies and nitrogen atoms have been previously reported to boost the pseudocapacitive behavior of metal oxides.^6,19^

Furthermore, the presence of pseudocapacitance and energy storage stands out, observed in Figure 7C, which illustrates the relationship between the peak current (*I*_p_) in the anodic and cathodic directions versus the square root of the sweep speed (υ^1/2^). This graph offers a clear view of the linearity present in the samples, which points to the influence of the diffusion process in controlling mass transport and a correlation coefficient (*R* ^2^) equal to 0.99.^54,55^ Detailed values of *R* ^2^ can be obtained from Table S1.

The galvanostatic charge and discharge (GCD) curves for both calcined and nitrogen-doped Nb_2_O_5_, in a potential range of 0.0–0.43 V, are illustrated in Figure 8. The interpretation of these curves allowed the specific capacity (*Q*) to be calculated using eq 4. The *Q*, specific capacitance (*C*_s_), and corresponding Coulombic efficiency values are listed in Table 2. Nb_N presented a specific capacity of 1297.37 C g^–1^, while Nb_U had 1108.75 C g^–1^ at a current density of 1 A g^–1^. Although there is a difference in active mass deposited on the electrodes (0.011 g for Nb_U and 0.0021 g for Nb_N), the calculation of specific capacity is normalized by the effective active mass, allowing a direct comparison between the materials. For the corresponding Coulombic efficiencies, obtained through the application of eq 6, Nb_U presented 70.96% at a current density of 1 A g^–1^. From 5 A g^–1^ onward, both materials present a Coulombic efficiency higher than 96%.

**Table 2:** Specific Capacity, Specific Capacitance, and Coulombic Efficiency for Nb_U and Nb_N Electrode Materials at Different Current Densities

	specific capacity (C g^–1^)		specific capacitance (F g^–1^)		Coulombic efficiency (%)	
current density (A g^–1^)	Nb_U	Nb_N	Nb_U	Nb_N	Nb_U	Nb_N
1	1108.75	1297.37	2578.49	3017.14	70.96	80.78
2	916.99	969.05	2132.55	2253.61	75.36	94.20
5	707.50	884.42	1645.35	2056.80	96.43	96.81
10	537.49	725.00	1249.99	1686.05	100	97.37
20	394.99	530.24	918.60	1233.11	100	96.43

**Figure 8:**
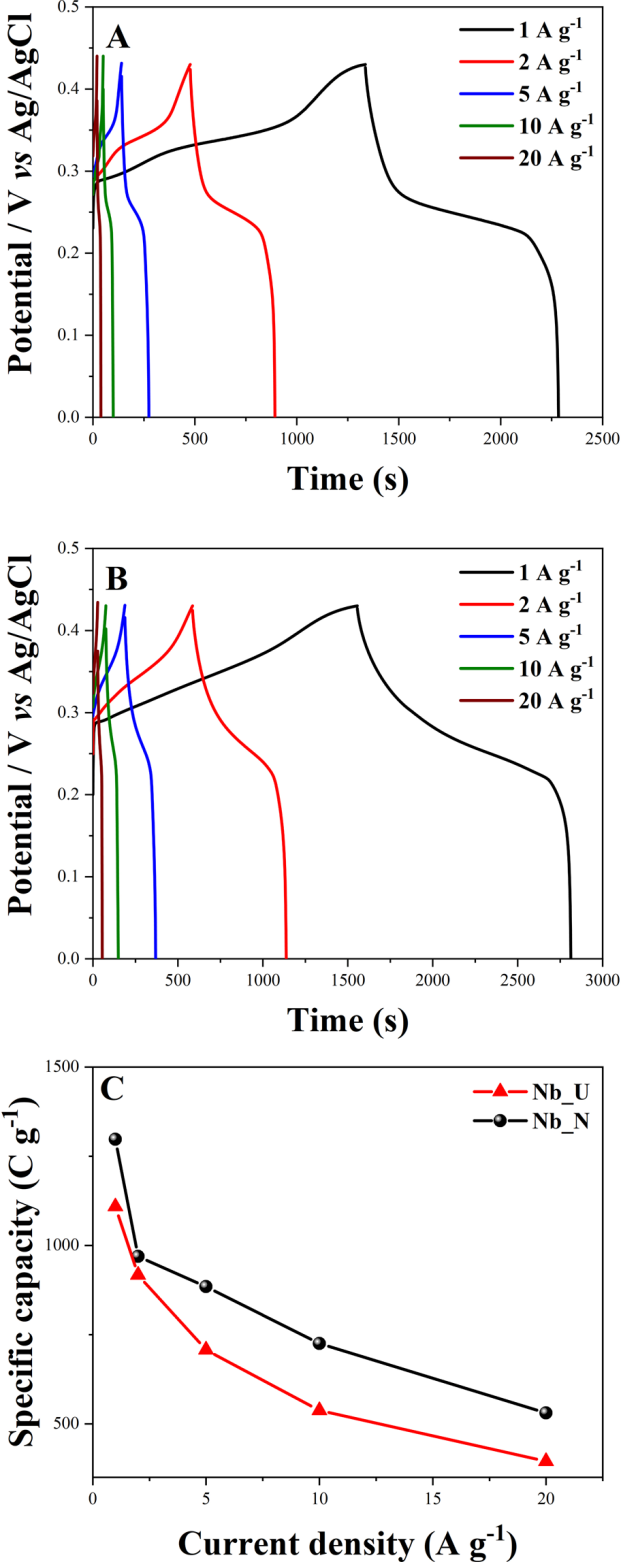
Charge and discharge curves (GCD) for Nb_U (A) and Nb_N (B) at different current densities and specific capacity versus current density (C).

The GCD curves of Nb_N (Figure 8B) show longer charge–discharge times than Nb_U (Figure 8A) at all current densities, indicating improved energy storage and rate capability. This enhancement arises from nitrogen doping, which increases conductivity and surface accessibility, facilitating charge transfer and ion diffusion. Even at high current densities, Nb_N maintains extended charge–discharge durations, confirming its superior electrochemical performance.

Figure 8C shows the specific capacity as a function of current density for both electrode materials. The *Q* values of Nb_N are higher than those of Nb_U across all current densities. Moreover, Nb_N exhibits a slower decline in specific capacity with increasing current density, demonstrating superior rate capability and overall electrochemical performance. As the current density increases, the specific capacity of both materials decreases, a common phenomenon in supercapacitors. This reduction is primarily due to limitations in ionic diffusion at higher currents, resulting in incomplete ion adsorption and hindered surface redox reactions at elevated scan rates.^56^ Thus, the cation accumulation process occurs at a slower rate, allowing active sites and accessible pores to participate in the energy storage mechanism.

The results obtained for the Nb_N material proved to be promising for its application in energy storage. Furthermore, we were able to compare our results with some materials already reported in the literature, as shown in Table 3. Several factors contribute to the enhanced performance of Nb_N. Nitrogen doping enhances the conductivity and electrochemical active sites, improving charge storage. Moreover, the use of a KOH electrolyte may facilitate better ion transport and interaction with the electrode. The morphology of “round and angular particles” provides a high surface area and active sites for charge storage, which could explain its superior specific capacity. The presence of graphitic carbon, in round particles, as seen in Raman results can also improve electrochemical performance.^37^

**Table 3:** Specific Capacitance of Nb_N in Comparison with Recent Works^a^

material	structure	synthesis	electrolyte	*C*_s_	current density (A g^–1^)	ref.
Nb_N	nanoparticles	urea-assisted doping	2 M KOH	(1303.90 C g^–1^) 3017.14 F g^–1^	1	this work
Nb_2_O_5_@Gr	nanocomposite	electrospray	1 M Li_2_SO_4_	150 μF cm^–2^	0.6	^57^
H–Nb_2_O_5_/rGO	nanocomposite	hydrothermal	LiClO_4_	190 mAh g^–1^	10	^58^
N porous MXene (Ti_3_C_2_)	MXene nanosheets	electrospinning	2 M H_2_SO_4_	176 F g^–1^	2	^59^
CeO_2_–MnO_2_–NGr	nanocomposite	hydrothermal	1 M KOH	772 F g^–1^	1	^60^
HNbMoO_6_	nanosheet	solid-state reaction	H_2_SO_4_–PVA–Na_2_MoO_4_ (1:1:0.25 wt %)	670 F g^–1^	1	^61^
Nb_2_O_5_@AC–CNT	nanocomposite	electrospray	1 M Li_2_SO_4_	232 F g^–1^	5	^62^
T- Nb_2_O_5_	orthorhombic	hydrothermal	1 M LiPF_6_	209 mAh g^–1^	1	^63^
Nb(PDCA)MOF@CNF	nanocomposites	hydrothermal	LiClO_4_	321 F g^–1^	10	^64^
Nb_0.06_NS	nanoflake	hydrothermal	2.0 M KOH	1482 F g^–1^	1	^65^
N-TiO_2_	nanoparticles	sol–gel	3.0 M KCl	311 F g^–1^	1	^66^
MnO_2_/HCS	nanoparticles	hydrothermal	1 M Na_2_SO_4_	255 F g^–1^	1	^67^
N,OPC-ZnK	nanosheet	pyrolysis	6 M KOH	271.4 F g^–1^	1	^68^
Nb_2_O_5_/g-C_3_N_4_/PPy	nanocomposite	in situ polymerization	1 M H_2_SO_4_	668.87 F g^–1^	1	^69^
BiNbO_4_@rGO	nanocomposite	hydrothermal	2 M KOH	1045.13 F g^–1^	1	^70^
NbSe_2_/rGO	nanocomposite	hydrothermal	2 M KOH	1512.1 F g^–1^	1	^71^

a Gr = graphene; N-doped = N; porous carbon = PC; HCS = hollow carbon spheres.

Cycling stability tests are essential for evaluating the long-term durability and performance retention of supercapacitors during repeated charge–discharge cycles. Thus, cycling stability tests were conducted at a current density of 20 A g^–1^ over 1000 charge and discharge cycles for both Nb_U and Nb_N. For comparison with previous studies on Nb_2_O_5_-based electrode materials, Figure 9 presents the variation of *C*_s_ with the cycle number. After 200 charge and discharge cycles, Nb_U exhibits a reduction of 53.3% in specific capacitance, reaching 372.09 F g ^–1^, and by the end of 1000 cycles, the capacitance has further declined to 40% (279.07 F g ^–1^) of its initial value. These results suggest that Nb_U undergoes degradation, loss of active material, or secondary reactions during cycling.^72^ In contrast, Nb_N maintains a specific capacitance that is 67.76% higher than that of Nb_U, with a capacitance retention of 33% after 1000 charge and discharge cycles. The higher capacitance values observed in Nb_N compared to Nb_U indicate that nitrogen doping likely enhances energy storage performance during GCD cycles.^73^

**Figure 9:**
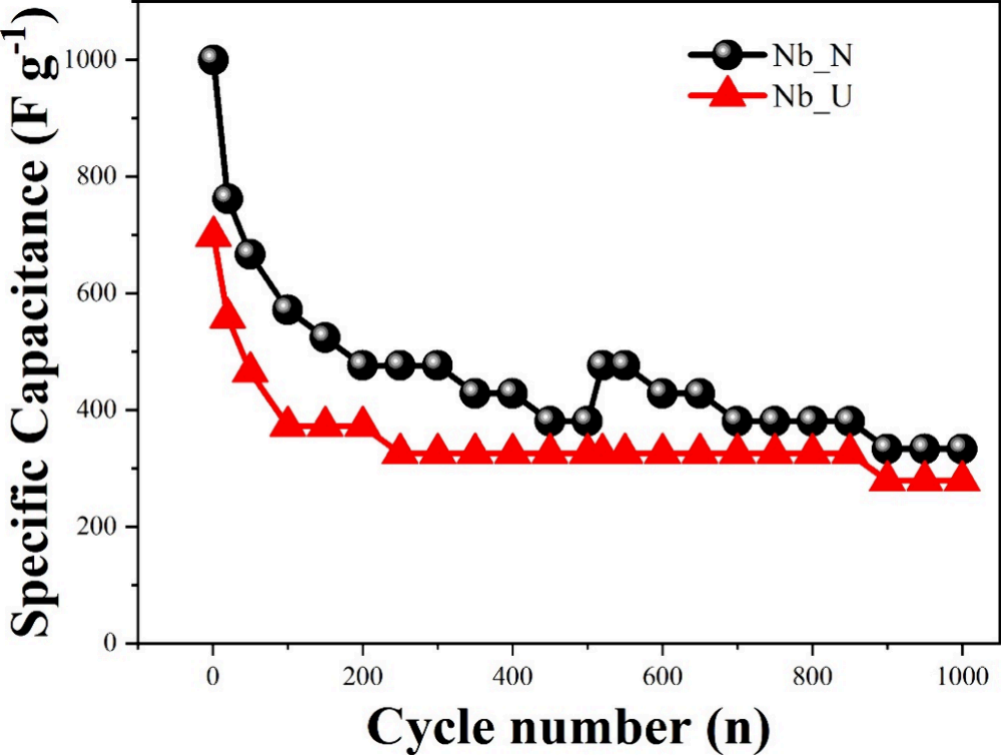
Specific capacitance during 1000 GCD cycles for Nb_N and Nb_U.

The Nyquist plots for the Nb_U and Nb_N electrode materials are presented in Figure 10. Electrochemical impedance spectroscopy (EIS) analyses were conducted under open-circuit potential, in a 2.0 M KOH electrolyte, both before and after 1000 and 2000 cycles, for Nb_U and Nb_N, respectively. The Nyquist graph is composed of the imaginary part (−*Z*″) and the real part (*Z*′). The formation of a semicircle in the high-frequency region is evident, representing charge transfer at the interface between the electrolyte and the oxide.^74–76^

**Figure 10:**
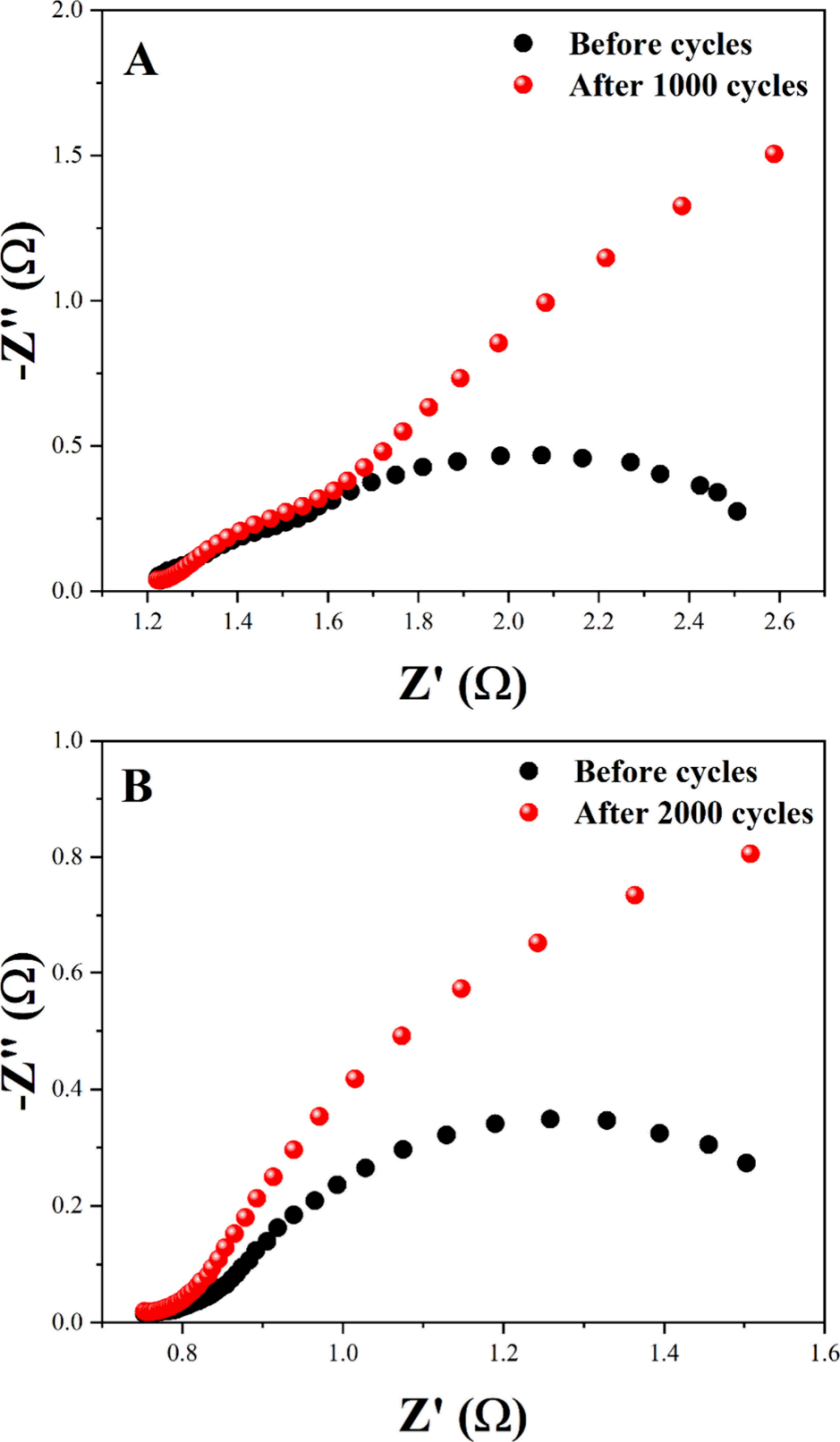
EIS before and after 1000 cycles (A) and EIS before and after 2000 cycles (B) of CDG for Nb_U and Nb_N, respectively.

The series resistances (*R*_s_) of the Nb_U and Nb_N materials remained stable over the device’s life cycle, measuring 1.8 Ω and 878 mΩ before and after 1000 galvanostatic charge–discharge (GCD) cycles, respectively. The stable *R*_s_ values demonstrate that these materials maintain consistent electrical conductivity, reflecting their durability over prolonged cycling.^77^

Conversely, the polarization resistances (*R*_p_) exhibited distinct variations between the two materials. For the Nb_U electrode material, the *R*_p_ varied between 1.29 Ω before and 5.85 Ω after 1000 GCD cycles, while for Nb_N, 807 mΩ was recorded before and 2.20Ω after 1000 GCD cycles. These variations indicate differences in the electrode–electrolyte interface properties between the two materials, influencing their charge storage capacity and the efficiency of the electrochemical process. Changes in *R*_p_ resistances can be attributed to greater charge transfer resistance. The equivalent circuits are depicted in Figure S2.

### 3.3 AC//Nb_N Supercapattery

A supercapattery combining Nb_N as a positive electrode and activated carbon (AC) as a negative electrode was investigated for energy storage performance, with total active masses of 0.9 mg of Nb_N and 4.2 mg of activated carbon (AC). Electrochemical characterization was conducted using the CV technique with a constant scan rate of 5 mV s^–1^ in a 2 M KOH solution, in a potential window of −1.2 to −0.0 V for AC and −0.2 to 0.5 V to Nb_N. Figure 11A presents the CV curves, highlighting the distinct electrochemical behaviors of the Nb_N and AC electrodes. The Nb_N electrode exhibits pseudocapacitive behavior, characterized by well-defined redox peaks, while the AC electrode demonstrates electrical double-layer capacitance (EDLC) with a nearly rectangular shape, which indicates rapid and reversible charge storage. These behaviors are observed within the potential windows of −0.2 to 0.5 V for Nb_N and −1.2 to 0 V for AC. This complementary behavior points to the synergy between AC and Nb_N in supercapatteries. Furthermore, the mass balance of the prepared positive and negative electrodes was determined using eq 4, allowing for a more comprehensive assessment of supercapattery properties and performance.^78,79^

**Figure 11:**
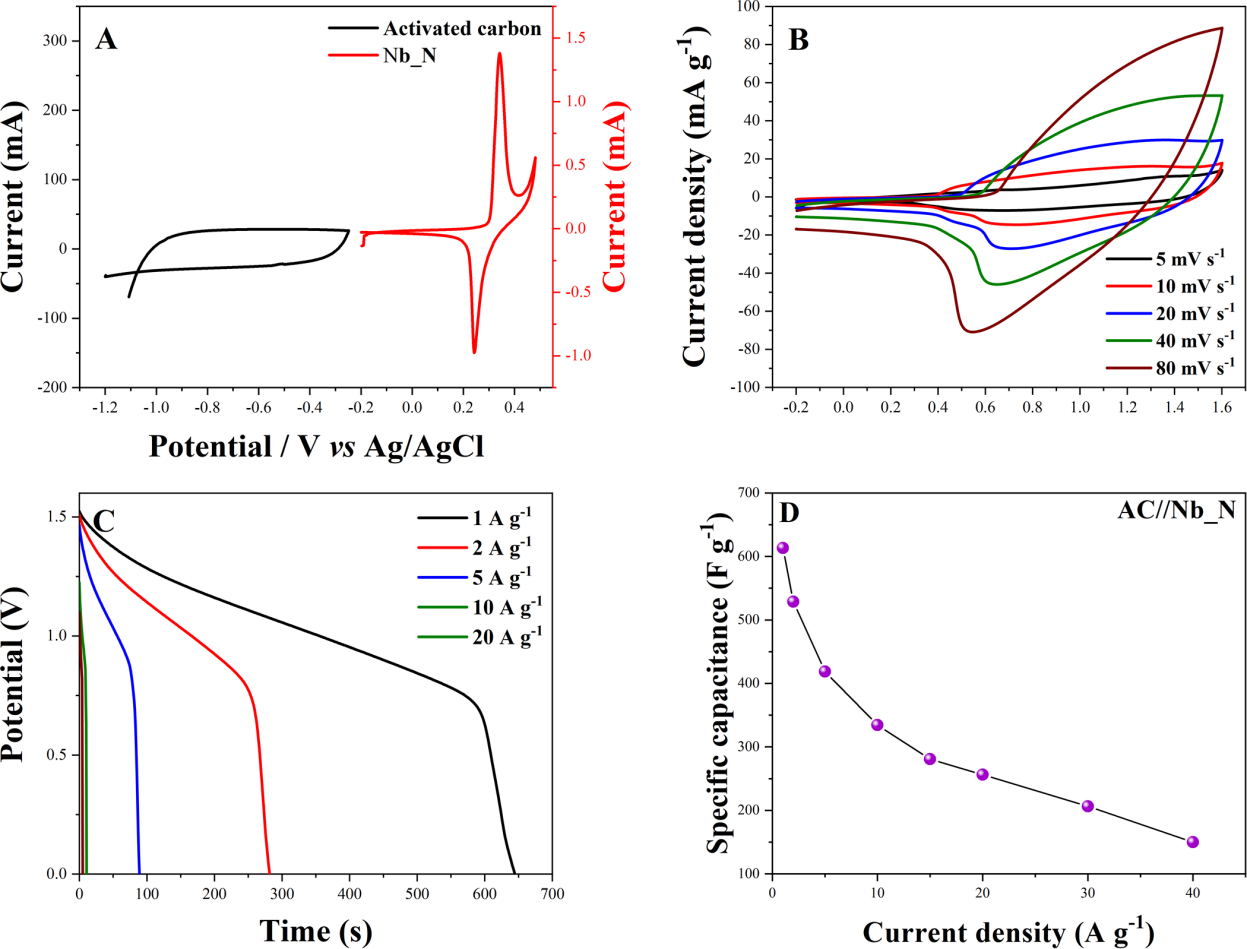
CV of AC and Nb_N at 5 mV s^–1^ in a three-electrode system (A). AC//Nb_N electrochemical measurements: CV at different scan rates (B), GCD at different current densities (C), and specific capacity versus current density (D).

In Figure 11B, the CV curves for the supercapattery AC//Nb_N are presented, at scanning speeds that varied from 5 to 80 mV s^–1^, in a potential window from −0.2 to 1.6 V. The CV curves at various scan rates retain their overall shape, demonstrating good capacitive behavior and charge transfer kinetics. At lower scan rates, the curves are well-defined, indicating efficient utilization of the electrode material. At higher scan rates, the distortion of the CV profile reflects a limitation in ion diffusion and charge transfer dynamics, a common phenomenon in hybrid energy storage devices.^80^ The performance of AC//Nb_N was analyzed at various current densities (1, 2, 5, 10, 20, 30, and 50 A g^–1^), as demonstrated in Figure 11C. The results revealed an initial specific capacity of 645 C g^–1^ at 1 A g^–1^ and a final specific capacitance of 50 C g^–1^ at a current density of 50 A g^–1^, as noted in Figure 11D. As in the three-electrode system (Figure 8C), capacitance decreased with increasing current density since at higher rates cations cannot fully access active sites, limiting storage capacity.

The AC//Nb_N supercapattery exhibited long-term electrochemical stability when tested at a high current density of 15 A g^–1^ (Figure 12). As in the three-electrode study, it is presented the variation of *C*_s_ with the cycle number to allow a direct comparison with previous works on Nb_2_O_5_-based materials. The specific capacitance initially increased from 68 to 113 F g^–1^ within the first 500 cycles, followed by stabilization in 90 F g^–1^, at 1000 GCD cycles. This indicates an activation process likely associated with progressive wettability, penetration of the electrolyte into previously inaccessible pores or active surfaces, and gradual stabilization of the electrode/electrolyte interface, as reported in previous works.^6,81,82^ After this stage, *C*_s_ stabilized and remained nearly constant for over 5000 cycles, demonstrating excellent cycling durability. Beyond this point, a gradual decline was observed, reaching 22 F g^–1^ after 10,000 cycles, which still reflects significant structural integrity under severe charge–discharge conditions. Nitrogen doping likely enhanced the structural resilience of niobium oxide, while the high surface area of activated carbon ensures reliable double-layer capacitance. AC//Nb_N achieved consistent performance over time, demonstrating applicability in energy storage.^77^

**Figure 12:**
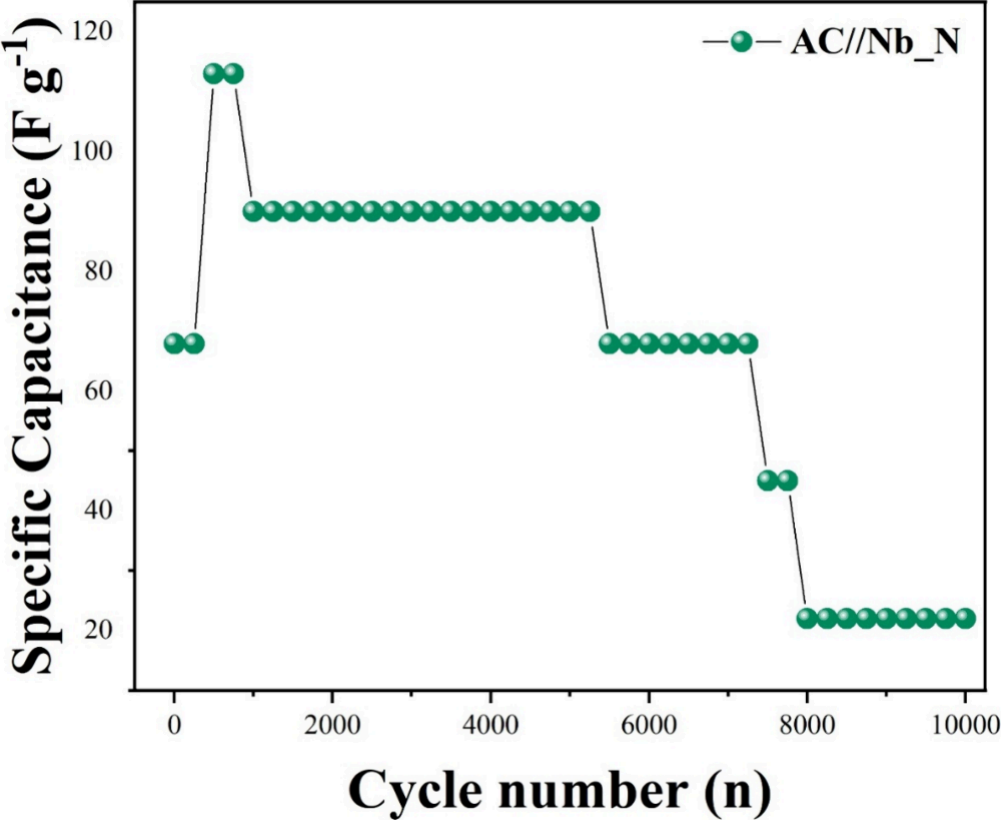
Specific capacitance during 10,000 GCD cycles at 15 A g^–1^ for AC//Nb_N.

The energy density (ED) and power density (PD) values for the hybrid device were calculated using eqs 5 and 6, respectively. These calculations resulted in a maximum energy density of 496.65 Wh kg^–1^ and a maximum power density of 2771.99 W kg^–1^. The results for AC//Nb_N were compared with results reported in the literature (Table 4 and Figure S3). The high ED and PD values demonstrate that this electrode material is suitable for high-capacity energy storage and rapid response to power demands.^83^

**Table 4:** Comparison of the AC//Nb_N Supercapattery with Similar Devices^a^

material	operating potential (V)	electrolyte	*C*_s_	power density (W kg^–1^)	energy density (Wh kg^–1^)	ref.
AC//Nb_N	1.8	2 M KOH	(645 C g^–1^) 418.83 F g^–1^	2771.99	496.65	this work
C-T-Nb_2_O_5_//AC	3.0	1 M LiPF_6_	34.8 F g^–1^	374.98	43.4	^63^
Nb(PDCA)MOF@CNF//CNF	1.7	1 M LiCl	272.1 F g^–1^	252.5	64.24	^64^
T-Nb_2_O_5_@Ni_2_P//AC	1.7	2 M KOH	28.8 mAh g^–1^	453	30.2	^84^
HNbMoO_6_//HNbMoO_6_	1.6	H_2_SO_4_–PVA–Na_2_MoO_4_ (1:1: 0.25 wt %)	190 F g^–1^	900	86	^61^
T-Nb_2_O_5_//AC	2.5	1 M Na_2_SO_4_	366 F g^–1^	650	86	^85^
Nb_2_O_5_/CNT//AC	3.0	1 M LiClO_4_	376 F g^–1^	82	33.5	^200^
NC//K_0.3_WO_3_	1.6	1 M H_2_SO_4_	222.4 F g^–1^	404.2	26.3	^86^
MnO_2_@bamboo leaf carbon//NC	1.6	1 M Na_2_SO_4_	32.25 F g^–1^	1600	11.47	^87^
PC@MnO_2_//NPC	1.8	1 M Na_2_SO_4_	78.2 F g^–1^	26.8	34.7	^88^
MnO_2_/HCS//HCS	2.0	1 M Na_2_SO_4_	74.5 F g^–1^	500	41.4	^67^
Co_0.85_Se//NPC	1.6	2 M KOH	125 F g^–1^	400	21.1	^89^
N,O PC-ZnK//N,O PC-ZnK@C	1.0	3 M KOH	46.6 F g^–1^	500	6.5	^68^
Ni/NGr CoMn_2_O//Ni/NGr	1.8	3 M KOH	(176.8 C g^–1^)	992.6	44.1	^90^
RuO_2_–NC//AC	1.5	2 M KOH	53 F g^–1^	751.66	16.71	^91^
Zn–Ni LDH@NiMoS_*x*_//ZIF-8 NC	1.6	2 M KOH	73.18 mAh g^–1^	549.13	58.54	^92^
NGr//MoS_2_/CdS	1.5	3 M KOH	107 F g^–1^	760	34	^93^

a N-doped carbon = NC; porous carbon = PC; N-doped graphene = NGr ; HCS = hollow carbon spheres.

## 4 Conclusions

In this work, nitrogen-doped niobium oxide (Nb_N) was synthesized via thermal treatment with urea. The characterizations confirmed successful nitrogen incorporation and structural modifications. Nitrogen doping effectively modified the physicochemical properties of Nb_2_O_5_, improving structural order and introducing oxygen vacancies as confirmed by XPS. The XRD, TEM, and Raman analyses evidenced lattice distortion and local disorder associated with nitrogen incorporation.

Electrochemical tests in 2 M KOH revealed that Nb_N exhibited superior performance compared Nb_U, achieving a specific capacity of 1297.374 C g ^–1^ at 1 A g ^–1^, excellent rate capability, and improved cycling stability. Nitrogen doping favored the formation of additional active sites and promoted faster faradaic reactions. The AC//Nb_N supercapattery delivered an energy density of 496.65 Wh kg^–1^ and a maximum power density of 2771.99 W kg^–1^, maintaining excellent stability over 5000 cycles. This study demonstrates that nitrogen doping can improve the electrochemical performance of Nb_2_O_5_, highlighting its potential for energy storage.

## Supplementary Material



## Notes

EDS spectra for Nb_U and Nb_N (Figure S1), correlation coefficients between anodic and cathodic directions for the electrode materials (Table S1), EIS equivalent circuits before and after 1000 CDG cycles (Figure S2), and Ragone plot (Figure S3) (PDF)
