# Supplementary material for: Enhanced Supercapattery Performance Enabled by Nitrogen-Doped Nb2O5 Nanostructures
Source: ACS Omega. 2025 Nov 26;10(48):59004–20. doi: 10.1021/acsomega.5c07882 (PMC12771455; doi:10.1021/acsomega.5c07882)
Supplement: Supplementary file 1 [file ao5c07882_si_001.pdf]

Supporting information

Enhanced Supercapattery Performance Enabled by Nitrogen-Doped Nb_2O_5 Nanostructures

Fernando José Soares Barros,[ab*] Samuel da Silva Eduardo,[c] Klebson Lucas Pereira Cardozo,[a] Hector A. Vitorino,[d] Carlos Martins Aiube,[e] Mariana Lumi Ichihara Sado,[f] Camila de Lima Ribeiro,[f] Alysson Martins Almeida Silva,[f] Francisco Murilo Tavares Luna,[b] Auro Atsushi Tanaka[a*]

[a] Department of Chemistry, Federal University of Maranhão, Av. dos Portugueses, 1966, 65080-805, São Luís, MA, Brazil.

[b] Department of Chemical Engineering, Federal University of Ceará, Campus do Pici, 709, Fortaleza, CE, 60.455-760, Brazil.

[c] Department of chemistry, Universidade Federal do Rio de Janeiro, Avenida Athos da Silveira Ramos, nº 149, 21941-909, Rio de Janeiro, RJ, 21941-901, Brazil.

[d] Department of Fundamental Chemistry, Institute of Chemistry, University of São Paulo, Av. Prof. Lineu Prestes, 748, São Paulo, SP 05508-000, Brazil.

[e]Institute of Chemistry, University of Brasília, Campus Universitário Darcy Ribeiro, Asa Norte, 70910-900, Brasília, DF, Brazil

[f]Department of Mechanical Engineering, University of Brasilia, Campus Universitário Darcy Ribeiro, Asa Norte, 70910-900, Brasília, DF, Brazil

*Corresponding authors: fernando.barros@gpsa.ufc.br , tanaka.auro@ufma.br

Table S1: The correlation coefficient between the anodic and cathodic directions for the Nb and Nb_N samples.

Samples	R^2	
	**Anodic**	**Cathodic**
**Nb**	0.9980	0.9979
**Nb_N**	0.9998	0.9981

R^2 correlation coefficient

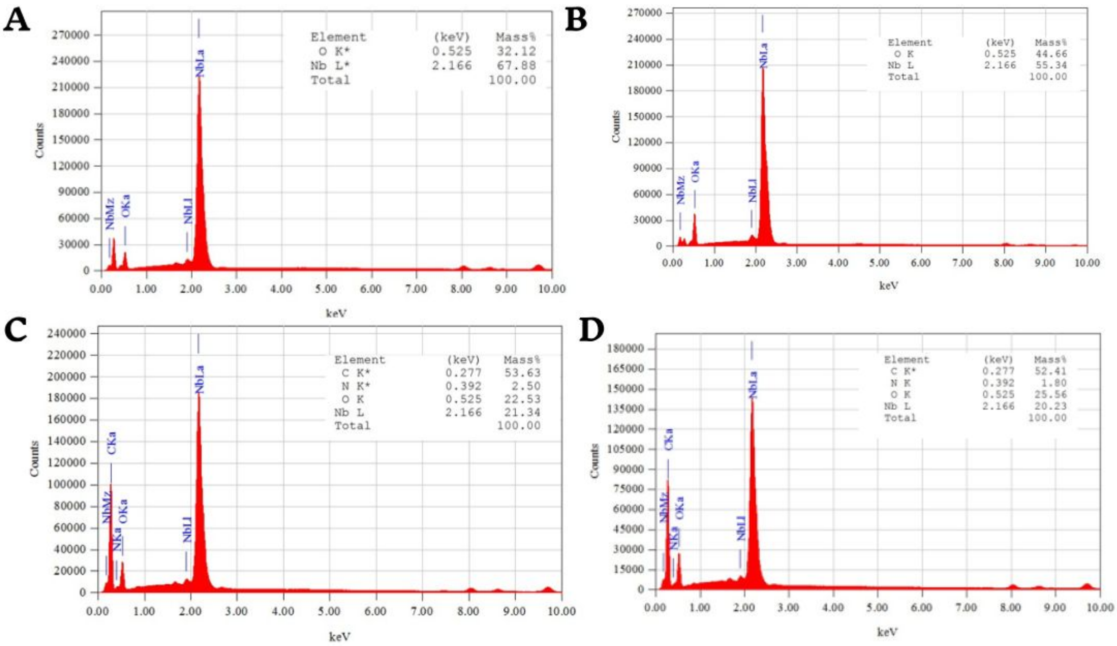

Figure S1: Energy dispersive spectroscopy (EDS) spectra of Nb_U angular (A) and round (B) particles. EDS spectra of Nb_N angular (C) and round (D) particles

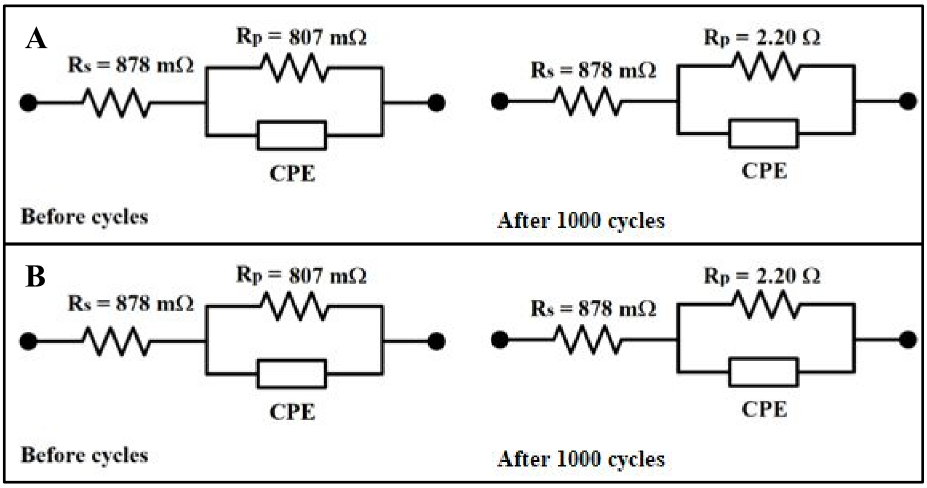

Figure S2: Equivalent circuit before and after 1000 CDG cycles for Nb (A) and Nb_N (B), respectively.

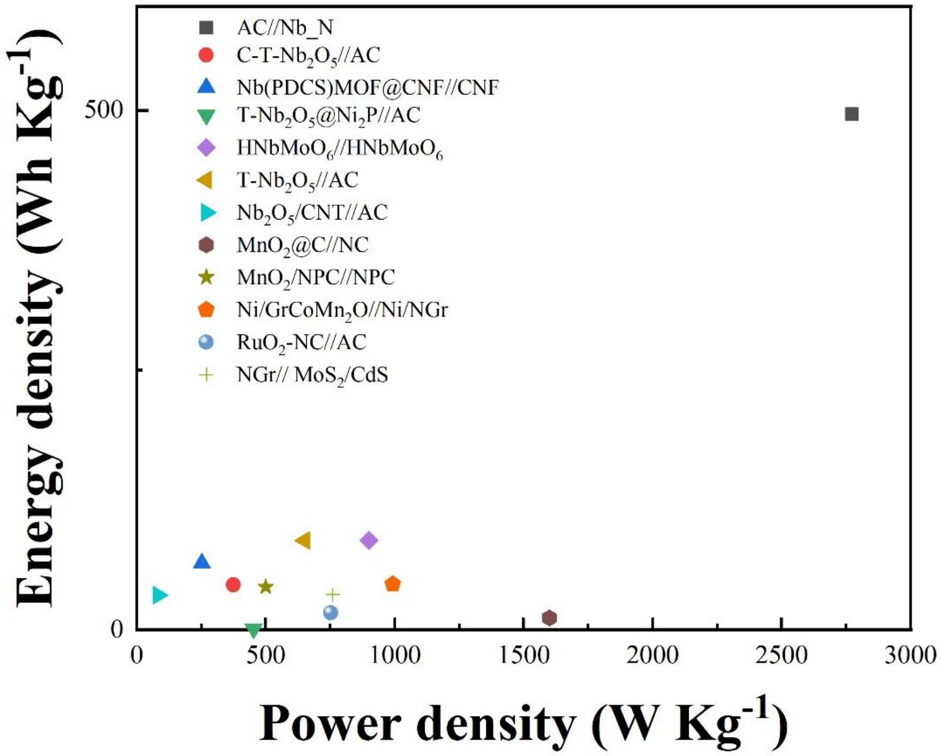

Figure S3: Ragone plot.
